# Future-proof Radiation therapist (RTT) practice in a pandemic – Lessons learnt from COVID-19

**DOI:** 10.1016/j.tipsro.2021.02.001

**Published:** 2021-02-05

**Authors:** Maeve Kearney, Mary Coffey, Maddalena Rossi, Yat Tsang, on behalf of the ESTRO ROSQC, RTTC

**Affiliations:** a Applied Radiation Therapy Trinity, Discipline of Radiation Therapy, School of Medicine, Trinity College, Dublin 2, Ireland; b Department of Radiation Oncology, The Netherlands Cancer Institute, Amsterdam, the Netherlands; c Radiotherapy Department, Mount Vernon Cancer Centre, Northwood, Middlesex, United Kingdom

**Keywords:** COVID-19, Radiotherapy, RTT

## Abstract

**Background and Purpose:**

The European SocieTy for Radiotherapy and Oncology Radiation Therapist Committee (ESTRO RTTC) published a guidance document and infographic providing recommendations to minimise risk of COVID-19 transmission in radiotherapy (RT) departments. The purpose of this study was to investigate the changes embedded in RT practice in the COVID-19 era and to recommend proactive measures to protect RT practice in future pandemics.

**Materials and Methods:**

The study was initiated by the ESTRO Radiation Oncology Safety and Quality Committee (ROSQC). A survey consisting of multiple choice, open ended and Likert scale questions was created to analyse the extent of changes embedded in RT practice in response to the COVID-19 pandemic under the four domains: patient care, RTT workflow, remote working and RT practice. This online survey was distributed globally in May 2020.

**Results:**

229 respondents across 27 countries completed the survey. 60% of respondents reported continuing/commencing RT in COVID-19 patients. Routine testing of patients and RTTs was not common. Split teams' procedures, hot linacs and separate entrances were implemented by 50% of respondents. Remote working was implemented for RT team members where face to face patient contact was not essential. Lack of staff, connectivity issues and lack of confirmed positive cases in the department were the main reasons cited for not implementing recommended measures.

**Conclusion:**

It is suggested that RT departments have responded to the COVID-19 pandemic and implemented certain changes in RT practice. RT departments should act now to implement recommended proactive measures to protect patients and RTTs – frontline healthcare workers.

## Highlights

• RT is an essential service that must continue despite the challenges posed by COVID-19.
• Our study suggests changes were implemented into RTT practice in response to COVID-19.
• Proactive measures are needed to protect both RTTs and patients in future Covid surges.

## Introduction

COVID-19 is a highly infectious coronavirus first reported in December 2019 and characterised by the World Health Organisation (WHO) as a global pandemic in March 2020. By July 2020, over 14 million COVID-19 cases were confirmed worldwide with more than 600,000 deaths [1]. The virus has serious implications for people’s health and is imposing difficult challenges to general healthcare services, including radiation oncology.

Radiotherapy (RT) plays a key role in cancer management, and is recommended as part of treatment for more than 50% of cancer patients [2]. Radiation therapists (RTTs) play a key and irreplaceable role in cancer patients’ treatments. As frontline healthcare workers, they are at risk of exposure to COVID-19, and of transmitting the virus to their patients. In response to threats imposed by COVID-19 in RT, the Radiation Therapist Committee (RTTC) of the European SocieTy for Radiotherapy and Oncology (ESTRO) prepared a guidance document [3] and an infographic [4]. The RTTC represents RTTs at a European level within ESTRO addressing issues relevant to the work of RTTs in Europe and of finding and implementing means to advance the profession and raise the standard of care for patients. Detailed recommendations and guidance on necessary precautions to be adopted in routine clinical RTT practice were provided, with the purpose of ensuring that radiotherapy departments continue to provide a safe and efficient service to both RT staff and the public during the current COVID-19 pandemic [3], [4].

Healthcare resources are limited, with PPE (Personal Protective Equipment) shortages widely reported due to ongoing or recurrent COVID-19 outbreaks [5]. Until a significant percentage of the population are vaccinated, it is essential that contingency plans are in place for long-term RT service planning in anticipation of future outbreaks. Against this background, the RTTC in collaboration with the Radiation Oncology and Safety Committee (ROSQC) - first established in 2016 to address issues of safety and quality in treatment preparation and delivery and provide information and advice to ESTRO members - adopted a cross sectional study design using questionnaires with the aims (i) to investigate the extent of changes embedded in current clinical RTT practice internationally due to COVID-19; and (ii) to recommend potential proactive measures for future RTT practice to cope with further COVID-19 surges or potential future pandemics.

## Materials and methods

The study was initiated by the ESTRO ROSQC in April 2020. During the development stage of the survey, the study set its sample population as RTT professionals. The survey was built (with full permission) on ongoing work carried out by the Canadian Association of Provincial Cancer Agencies. It consisted of both demographic questions and questions covering the 4 topics addressed in the ESTRO RTT COVID-19 guidance document and Infographic: patient care, RTT workflow, remote working and RT practice [3], [4]. Open, multiple choice and Likert scale questions were used, and the details are listed in supplementary material 1. A distinction was made between High Income Countries (HIC) and Low to Middle Income Countries (LMICs) to define if resources in countries affected the decisions that were made. SurveyMonkey was used to conduct the survey.

The questionnaire was circulated to RTTs identified from sources including the ESTRO RTT membership database and via a link to the survey posted on the American Society of Radiologic Technologists (ASRT) member forum and World Wide Radiation Therapists Facebook page. Participants were asked to complete the survey within three weeks with a reminder email sent 5 days prior to the closing date. After the closing date the raw data results of the survey were analysed by representatives from both ROSQC and RTTC.

## Results

Between the 22nd May 2020 and 12th June 2020, 229 RTTs across 27 countries completed the survey (Table 1). The highest number of responses were from USA (17.0%), Netherlands (14.0%), United Kingdom (13.1%) and Denmark (12.2%). To ensure anonymity, respondents were asked to state country rather than city of practice. Therefore, it is possible that results from some countries are representative of RTT practice from just one department. For the analysis, the following countries were categorised as LMICs: Bosnia and Herzegovina, India, Jordan, Nepal and Serbia.

**Table 1:** A summary of the RTT responses and the countries participating in the survey.

Countries	Number of responses	Total percentage (%)
Australia	4	1.7
Austria	4	1.7
Bahamas	1	0.4
Belgium	12	5.2
Bosnia & Herzegovina	2	0.9
Canada	6	2.6
Denmark	28	12.2
Estonia	1	0.4
France	2	0.9
India	6	2.6
Ireland	12	5.2
Israel	1	0.4
Italy	4	1.7
Jordan	1	0.4
Malta	2	0.9
Nepal	1	0.4
The Netherlands	32	14.0
New Zealand	3	1.3
Norway	1	0.4
Poland	3	1.3
Portugal	17	7.4
Qatar	1	0.4
Serbia	2	0.9
Spain	2	0.9
Switzerland	10	4.4
United Kingdom	30	13.1
United States	39	17.0
Unknown	2	0.9

### Patient care

**Fig. 1:**
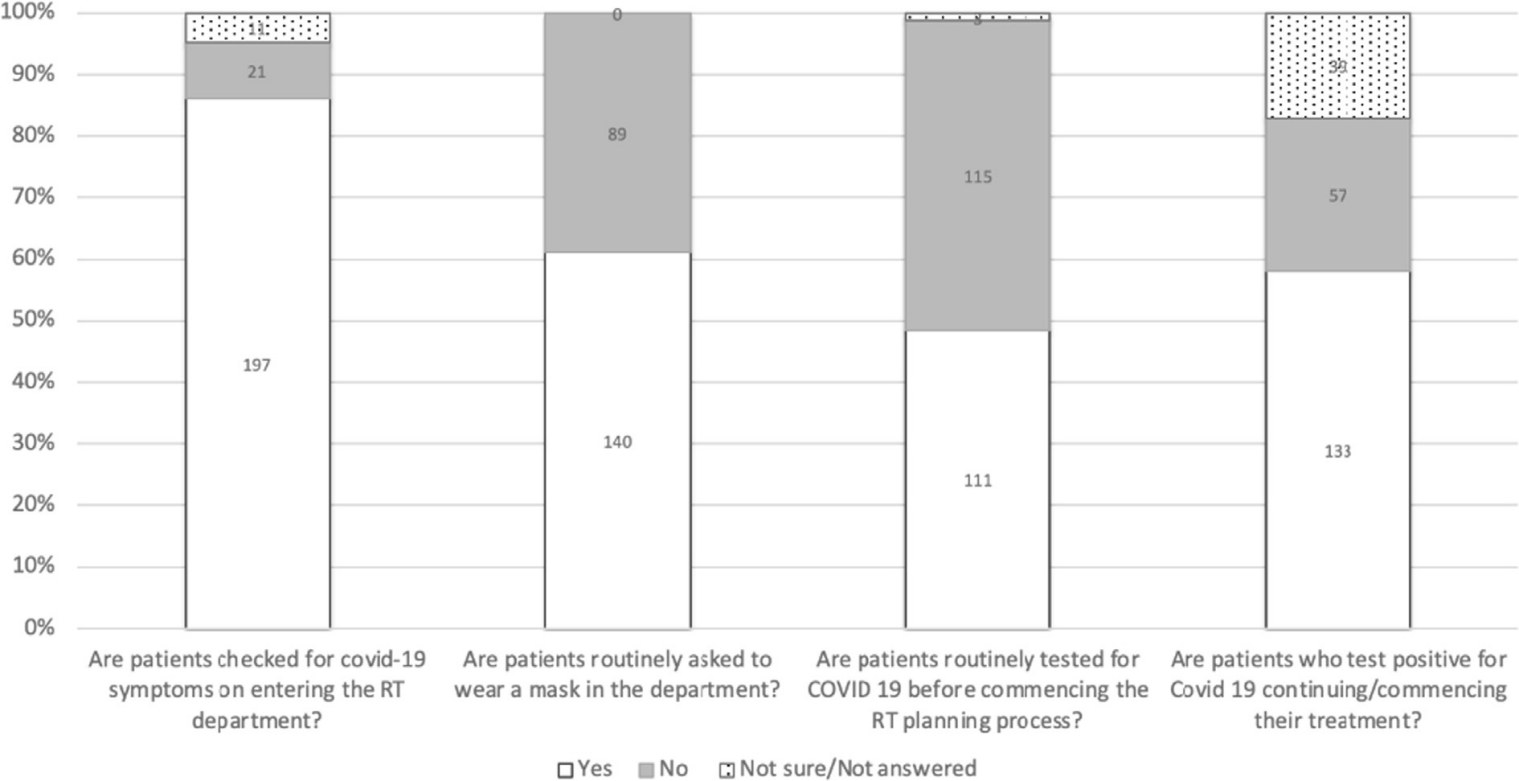
A bar chart illustrating the RTT responses to the survey under the domain of patient care.

82% of respondents stated that patients were checked for COVID-19 symptoms before entering the department, more than 60% of responses (140/229) suggested that patients were routinely asked to wear a mask in the department, less than half of the respondents (111/229) said that patients would be routinely tested for COVID-19 before commencing the radiotherapy planning process (Fig. 1). 71 of those 111 responses stated that COVID-19 testing prior to radiotherapy planning would only be carried out in certain groups of patients i.e. symptomatic patients. There was no indication in any responses that ongoing testing of patients throughout the treatment duration was being performed.

When the RTTs were asked if patients who tested positive for COVID-19 would be commencing/continuing their treatment, nearly 60% (133) of respondents answered “Yes” 39 responses stated “Not sure”, 19 of them stated that they would not know the answer for this question because they had no positive COVID-19 cases at the time of survey. Reviewing the responses from LMICs, 92% of RTTs stated that COVID-19 positive patients would not commence or continue radiotherapy in their departments.

### RTT workflow

**Fig. 2:**
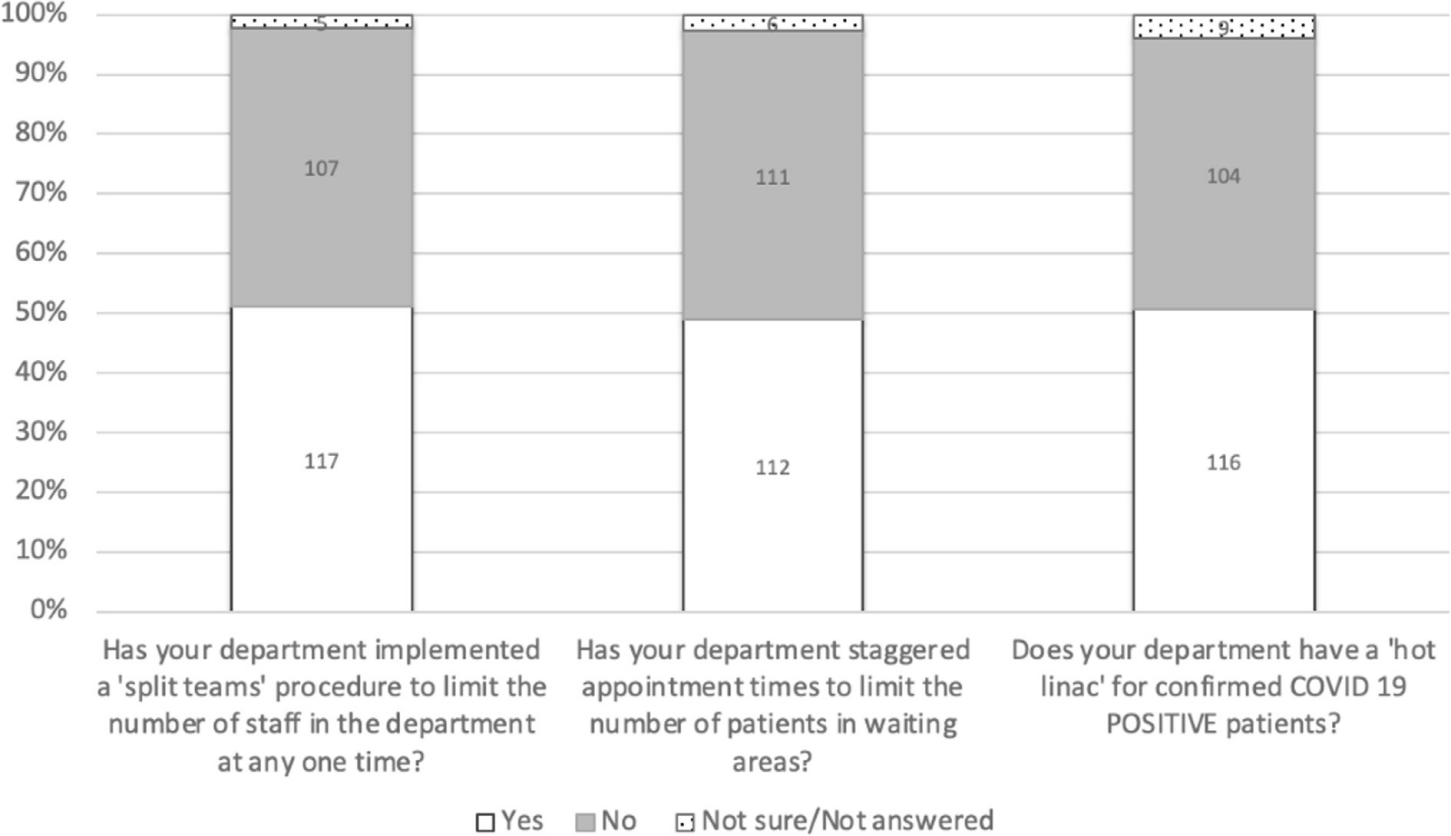
A bar chart demonstrating the RTT responses to the survey under the domain of RTT workflow.

On RT workflow procedures adopted for COVID-19 approximately half of the respondents stated their departments implemented approaches such as “split team” (51%), “staggered appointment times” (49%) and “hot linac” (51%) to minimise the number of patients/staff in the department at the same time and avoid potential exposure and transmission across the entire RT team (Fig. 2). Reviewing the responses from LMICs, nearly three-quarters reported they implemented all three approaches.

**Fig. 3:**
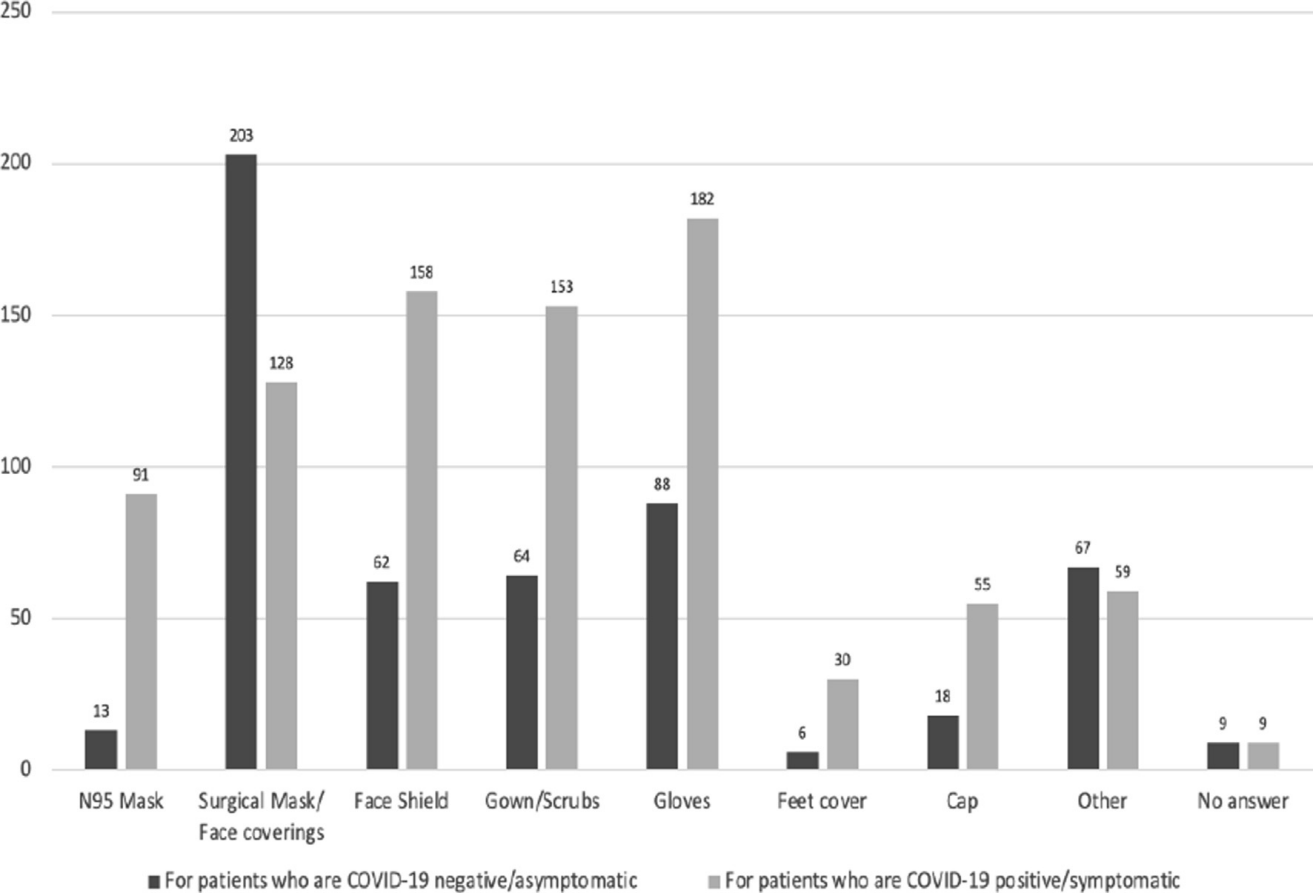
A bar chart summarising the RTT responses to the survey about the personal protection equipment for RTTs.

Masks and gloves were the most common types of PPE used by RTTs regardless of patient COVID-19 status. A greater variety and higher frequency of PPE use was reported by RTTs for COVID-19 positive/symptomatic patients. N95 masks were used more frequently for COVID-19 positive/symptomatic patients (Fig. 3).

Nearly three quarters of the respondents were satisfied with the quality of the PPE used for both COVID-19 positive/symptomatic (73%) and COVID-19 negative/asymptomatic (76%) patients in their department.

### Remote working

**Fig. 4:**
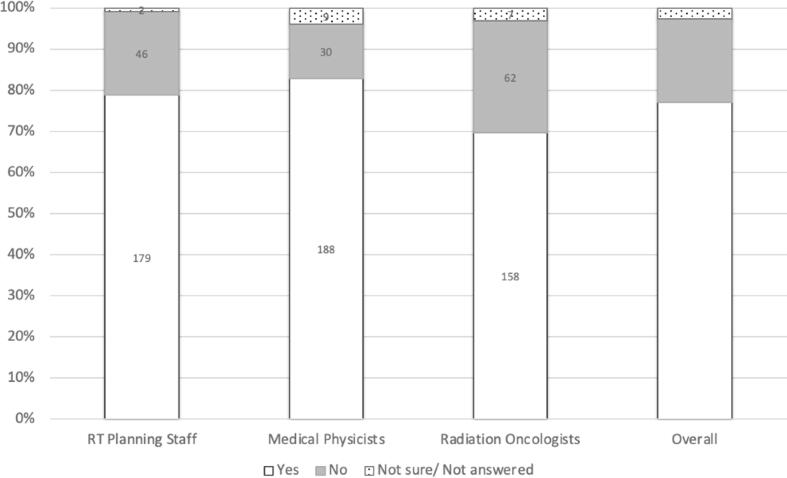
A bar chart showing the RTT responses to the survey under the domain of remote working.

Over three quarters of responses reported opportunities for clinical staff to work remotely due to COVID-19 with remote working implemented for medical physicists (87%), treatment planning staff {RTTs and dosimetrists} (75%), and radiation oncologists (67%) (Fig. 4). Over 80% of responses stating that remote working was not possible were from LMICs demonstrating a difference in practice in this setting. In some cases, remote working had only been allowed for one month during the peak of the pandemic.

Overall respondents reported a low level of stress {average 3.1 (1–10)}, on not having physical access to staff members who were working remotely; and it was reported as straightforward to contact radiation oncologists (71%), medical physicists (84%) and treatment planning staff (80%) who were working remotely to query clinical issues.

### RT practice

Routine screening of RTTs for COVID-19 was not common practice, with the majority (73%) of respondents stating ‘No’ (Table 2). To minimise the risk of infectious disease transmission, separate or COVID-19 specifically adopted entrances and waiting rooms (55%), and Perspex screens/shields in control areas (39%) were often implemented. Although there is evidence that COVID-19 may be detected on CBCTs in patients who were otherwise asymptomatic [6], [7], [8], [9], [10] nearly 80% of respondents stated ‘No’ when asked if suspicious COVID-19 related changes were detected on CBCT during routine clinical practice.

**Table 2:** A summary of the RTT responses to the survey under the domain of RT practice.

Questions	Number of responses (%)		
Are RTTs routinely screened for COVID-19 in your RT departments	Yes	No	Not sure/Not answered
	54 (24%)	167 (73%)	8 (3%)
Has your department implemented any of the following measures to minimise transmission?	Perspex screens	Separate/COVID-19 specifically adopted entrance and waiting room	Not sure/Not answered
	89 (39%)	126 (55%)	14 (6%)
Have you detected any suspicious COVID-19 related changes on CBCT during routine clinical practice?	Yes	No	Not sure/Not answered
	32 (14%)	178 (78%)	19 (8%)

## Discussion

COVID-19 has had a severe effect globally on healthcare service delivery. Initial studies attribute a significant decline in the numbers of cancer patients being referred for treatment to; the reduction in cancer screening, patients not presenting to their primary care providers with suspicious symptoms [9], [10] and delaying RT in low-risk patient groups such as radical prostate cancer RT [8]. These factors have a secondary impact with increased demand anticipated for RT services in the future [11]. As society reopens and adjusts to living with COVID-19, RT departments must learn to adapt to this new norm. The primary aim of this study was to establish the extent of changes embedded in current clinical RTT practice internationally due to COVID-19 and to make recommendations based on findings for future best practice. To our knowledge, our study is the first to provide actual survey data to examine the influence of COVID-19 on RT practice.

### Patient care

Cancer patients are considered a high-risk group for contracting COVID-19 [12], and the risks of contracting COVID-19 versus cancer disease progression must be carefully considered before delaying RT. International guidelines recommend prioritising RT in patients with rapidly proliferating tumours, emergency palliative patients and patients where RT was the primary treatment modality [13], [14], [15]. Nagar and Formenti contended that RT should not only continue to be used but its increased use should be considered as it does not compete for resources necessary to treat COVID-19 [11]. Despite this 92% of respondents from LMICs stated that COVID-19 positive patients were not starting/continuing RT while 8% of respondents were not aware of local policy as no positive cases had been reported in their department.

Measures recommended in the ESTRO guidance document such as symptom checks and mandatory mask wearing for patients [3], [4] were implemented into routine RTT clinical practice to a certain extent (Fig. 1). However, mask wearing was not enforced for all patients but in selected patient groups only e.g. head and neck patients, lung patients, patients who were symptomatic/COVID-19 positive or patients who recently travelled abroad. If mask wearing is not the norm this may lead to a sense of stigmatisation in patients. There is no doubt that guidelines with respect to physical and social distancing strike at the very core of our day-to-day norm [16] where compassion and a patient centred experience is key. Mask wearing conceals facial expressions making it more challenging to foster relationships potentially contributing to feelings of isolation that their disease may have already caused [17]. Some RTTs reported displaying their photo to add a personal touch to PPE that is otherwise impersonal.

Even though many patients attend as outpatients and are at risk of community transmission, symptom checks were carried out on their first visit to the RT department with no evidence of routine symptom checks throughout the treatment course.

*Recommendations:*

• *Mask wearing should be mandatory for all patients.*
• *Daily triage system should be in place for all patients entering the RT departments.*
• *Escalation procedures should be in place for patients that present with suspicious symptoms; testing procedures with swift turnaround and definitive guidelines for RTTs on which patient groups must commence/continue RT if tested positive.*

### RTT workflow

The ESTRO RTT guidance document recommended measures such as splitting teams and staggering patient appointment times to limit critical staff exposure and patient transmission [3], [4]. Nearly half the respondents reported it was not possible to implement split team shift patterns with staffing levels listed as the main barrier. Worryingly, some RT departments reported they were not permitted to stagger patient appointments as they would incur overtime costs. A designated ‘hot linac’ for confirmed COVID-19 positive cases was not common. ‘A lack of positive patients’, ‘treating patients at the end of the day’ and ‘specialised techniques such as Cyberknife, Tomotherapy’ reported as the main rationale for not having a dedicated ‘hot linac’.

Managers should be proactive and independently evaluate the potential effect of a further surge or future pandemic on their staff and how best to organise the workforce to ensure continuity of care. RTTs consistently voiced concerns on the anticipated increase in demand for RT services in the future and if their departments would cope. Delaying patients may not be an option if they have already experienced delays in the treatment process during the initial COVID-19 surge. Anderson et al gives detail of how they addressed workforce issues in his publication, and this provides an excellent guide to good practice in a pandemic setting [16].

WHO recommended PPE (mask, face shield, gloves, gowns) was routinely adopted into routine clinical RTT practice for COVID-19 positive/symptomatic patients (Fig. 3) [3]. However, N95 masks were not always used for this group of patients. Evidence shows that 50–80% of COVID-19 carriers may be asymptomatic [18], [19], yet mask wearing for RTTs in general was less common when treating patients who were asymptomatic. As RT is largely an out-patient procedure with routine testing reported as limited to patients who are symptomatic or due to be admitted, it is prudent to provide adequate PPE to treat all patients as potential disease carriers. While RTTs were generally satisfied with the quality of PPE available some reported clashes with management where they did not feel adequately protected and national guidelines for PPE use were based on supply rather than clinical evidence. Some respondents reported RTTs were asked to wear the same mask for up two weeks due to severe shortages of PPE in their countries. The emotional impact of COVID-19 on RTTs should be considered. RTTs may experience fear for themselves and their families which, interestingly, heightens the perception of risk and increases safety behaviour but, conversely, anger can lead to poorer consideration of how to best manage risk [12], [13]. To reduce stress and anxiety a basic requirement is the provision of sufficient suitable PPE.

*Recommendations:*

• *Implementing team pods/split teams is recommended to avoid a situation where an entire RTT team would have to self-isolate resulting in cessation of all RTT services within that department.*
• *Adequate time for infection control measures between patients should be allowed when scheduling patient appointments. The use of catch-up slots in RT appointment scheduling could be considered.*
• *RTTs should be provided with adequate PPE for treating all patients regardless of COVID-19 status.*

### Remote working

Government directives recommend implementing a work from home policy wherever possible to minimise social interactions. RT is an essential service and as RTTs are frontline workers - remote working can only be considered at limited stages of the patient pathway such as treatment planning and review clinics [20]. Remote working was reported among RT groups - treatment planning staff (RTTs and dosimetrists), medical physicists and radiation oncologists where face to face patient contact might not be essential. Our data cited connectivity issues, licensing issues and staffing levels (e.g permitted for a fixed period only at the height of the ‘peak’ and some treatment planning RTTs cover in treatment delivery also), as barriers to remote working. In some cases, radiation oncologists worked on site but conducted consultations virtually to reduce patient access to RT departments. Transferring face to face consultations to remote setting is implemented in several RT departments globally [21]. While this measure minimises social interactions, our respondents expressed concerns that symptoms may be missed due to lack of visual cues with poor connections.

Remote working in RT department future workforce planning, must be carefully considered. RTT practice is ever changing with an interdisciplinary approach essential to develop departmental protocols and processes. Research suggests that lack of face to face contact can reduce innovation and knowledge transfer in a workplace [22], [23] while RTTs cite face to face as the most efficient and effective mode of communication with the interdisciplinary team [24]. It is noted that further investigations could be carried out on if RTTs appreciate the flexibility and benefits of virtual online communication during the COVID-19 pandemic. If remote working for radiation oncologists, medical physicists and treatment planning staff continues, roles of RTTs should be revised with an emphasis on maximising autonomy in treatment delivery decision making skills. Appropriate RTT training and competency frameworks should be in place to maintain efficiency in all RT multidisciplinary processes.

*Recommendations:*

• *Impact of remote working on practice development in RT departments should be carefully considered and implemented where appropriate*
• *Decision making facilities of RTTs should be carefully considered if remote working remains standard practice in other RT groups*

### RTT practice

Without significant vaccination coverage, preventive measures to limit disease transmission should be proactive rather than reactive to accommodate increasing patient numbers under the pandemic. Our data suggested that measures for reducing transmission (Perspex screens and separate entrances for COVID-19 positive patients) recommended in the ESTRO guidance document [3], [4] were not widely implemented. As confirmed COVID-19 cases and overall workload was low, respondents felt there was little justification for these measures.

Some RT departments were operating a no waiting area policy with patients asked to wait in their cars until their appointment time and not have anyone accompany them into the RT department. This may isolate cancer patients and cause considerable psychological distress [17]. The important role of RTTs in caring and supporting patients at a time of crisis and distress has never been more important. RTTs should be recognised as frontline healthcare workers (direct physical contact with patients is unavoidable in treatment set ups) and prioritised in staff testing. However, 73% of the responses suggested that RTTs were not routinely screened for COVID-19 and this should be addressed given the vulnerability of cancer patients to COVID 19 and the close proximity required between patient and RTT.

Less than 20% of the respondents reported seeing suspicious COVID-19 related changes on CBCT during RT treatment. This can be due to the lack of positive or symptomatic cases referred for RT. Respondents were not asked directly if these changes were detected on planning CT scans, but some responses indicated that RTTs had reported suspicious cases using planning CT images which were subsequently investigated. Some respondents did remark that CBCT image quality was too poor while some reported that it was not departmental practice for RTTs to interpret volumetric images in their department. Several publications discuss the increased use of hypofractionated radiotherapy during the COVID-19 pandemic [25], [26]. This shift in practice away from conventional fractionation necessitates RTTs to have a greater understanding of the principles underpinning hypofractionated radiotherapy practice [27], [28], [29]. Implementing effective and safe IGRT strategies where RTTs are at the forefront is necessary to reduce geometric uncertainties and discrepancies between planned and delivered doses is a must [30], [31], [32]. RTTs should be equipped with the skills of detecting changes on CBCT that could indicate COVID-19 or any other volumetric changes that could have dosimetric consequences and promptly intervene with appropriate patient management.

*Recommendations:*

• *Routine COVID-19 testing with swift turnarounds should be in place for RTTs.*
• *RT departments must review current RTT education/training and competency framework to ensure that staff are competent in IGRT execution and delivery.*
• *An escalation procedure should be in place for RTTs when* suspicious COVID-19 related changes *are detected on planning CT and CBCT scans.*

## Conclusion

COVID-19 will remain a part of our lives until a significant percentage of the population are vaccinated. RT is an essential service and one that does not compete with healthcare resources for the pandemic. Our study demonstrates that RT departments have responded to the COVID-19 by implementing a certain level of changes in RT practice, and recommends a list of potential proactive measures under the four domains of patient care, RTT workflow, remote working and RT practice for future RTT practice.

RTTs are frontline healthcare professionals, their relationship with patients is key to achieving optimum experience for patients. RT departments must consider the implications of potential further COVID-19 surges in service demands and implement proactive measures to protect RTTs and patients.

## Declaration of Competing Interest

The authors declare that they have no known competing financial interests or personal relationships that could have appeared to influence the work reported in this paper.
