# Supplementary material for: Future-proof Radiation therapist (RTT) practice in a pandemic – Lessons learnt from COVID-19
Source: Tech Innov Patient Support Radiat Oncol. 2021 Feb 5;17:18–24. doi: 10.1016/j.tipsro.2021.02.001 (PMC7862908; doi:10.1016/j.tipsro.2021.02.001)
Supplement: Supplementary Data 1 [file mmc1.docx]

**Supplementary Material - Questionnaire**

The ESTRO Radiation Oncology Quality and Safety Committee together with the ESTRO RTT Committee would like to evaluate screening of staff and patients and the use of Personal Protective Equipment (PPE). The following questionnaire has already been used by the Canadian Association of Provincial Cancer Agencies who have shared it with us and we hope, in addition to evaluating the situation across Europe, to be able to compare the situation in the Canadian and European settings. In this context we would appreciate it if you could take the time to answer the short questionnaire *(link to survey monkey?)*

1. In which country is your centre located? ___________________
2. In your department, are patients who test positive for Covid-19 commencing/continuing their course of RT?
   1. Yes
   2. No
   3. Not sure

Comments _(please specify)_______________

**SECTION 1:**  **As of TODAY: Precautions for symptomatic,** **COVID 19 negative patients:**

1. Are health care providers / practitioners routinely screened in your RT department?
   1. Yes
   2. No
   3. Not sure

Comments (please specify)______

1. Are patients asked to routinely wear a mask in the RT department?
   1. Yes
   2. No
   3. Not sure

Comments_(please specify)_____

1. Are Patients checked for symptoms when entering the hospital/department?
   1. Yes
   2. No
   3. Not sure

Comments (please specify) ______________

1. A. Are patients routinely tested for COVID 19 before commencing the RT planning process?
   1. Yes
   2. No
   3. Not sure

Comments (please specify) ____________

6.B. Please specify which categories or patients are routinely screened. Tick all that apply.

a. Patient with a specific cancer diagnosis (please list)

b. Older patients

c. Patients coming from another care facility

d. Patients with non-specific symptoms

d. Other (please specify)

**SECTION 2: PPE protocol for Radiation TherapisTs (RTTs)**

1. For which of the following patients are RTTs routinely using PPE? Tick all that apply:
   1. All patients
   2. Patients awaiting test results
   3. Asymptomatic patients awaiting testing
   4. Patients who have tested positive for Covid-19
   5. Other – please comment
2. For **all patients who are NOT COVID 19 positive and are NOT symptomatic,** what PPE are RTTs using in the *pre-treatment area (mould room, simulation process, etc*) (please choose all that apply)
   1. Type of mask (surgical, N95, other – dropdown list here)
   2. Face shield
   3. Gown/scrubs
   4. Gloves
   5. Feet cover
   6. Cap
   7. Other (free text)
3. For **all patients, who are NOT COVID 19 positive and are NOT symptomatic** what PPE are RTTs using on the *treatment machines*? (please choose all that apply)
   1. Type of mask (surgical, N95, other – dropdown list here)
   2. Face shield
   3. Gown/scrubs
   4. Gloves
   5. Feet cover
   6. Cap
   7. Other (free text)
4. For **COVID 19 positive or suspected patients,** what PPE are RTTs using in the *pre-treatment area (mould room, simulation process, etc)* (please choose all that apply)
   1. Type of mask (surgical, N95, other – dropdown list here)
   2. Face shield
   3. Gown/scrubs
   4. Gloves
   5. Feet cover
   6. Cap
   7. Other (free text)
5. **For COVID 19 positive or suspected** patients, what PPE are RTTs using on the *treatment machines* ? (please choose all that apply)
   1. Type of mask (surgical, N95, other – dropdown list here)
   2. Face shield
   3. Gown/scrubs
   4. Gloves
   5. Feet cover
   6. Cap
   7. Other (free text)
6. a. Describe the procedure for daily use of PPE by each RTT working on the treatment unit for all patients who are NOT COVID 19 positive and are NOT symptomatic (please choose all that apply)

| PPE type | New PPE for each patient | PPE not changed for each patient but changed every x number of patients (drop down with number of changes) | Reusing PPE wherever possible |
| --- | --- | --- | --- |
| Mask |  |  |  |
| Face Shield |  |  |  |
| Gown/Scrubs |  |  |  |
| Gloves |  |  |  |
| Feet covers |  |  |  |
| Cap |  |  |  |
| Other(______) |  |  |  |

12.b. Are you following the general government guidelines on the use of PPE?

a. Yes

b. No

c. Not sure?Other (Please specify) _________

13.a.Describe the procedure for daily use of PPE by each RTT working on the treatment unit for **COVID 19 positive or suspected patients** (please choose all that apply)

| PPE type | New PPE for each patient | PPE not changed for each patient but changed every x number of patients (drop down with number of changes) | Reusing PPE wherever possible |
| --- | --- | --- | --- |
| Mask |  |  |  |
| Face Shield |  |  |  |
| Gown/Scrubs |  |  |  |
| Gloves |  |  |  |
| Feet covers |  |  |  |
| Cap |  |  |  |
| Other(______) |  |  |  |

13.b. Are you following the general government guidelines on the use of PPE?

a. Yes

b. No

c. Not sure/Other (Please specify)_________________

1. Please provide some key points from general governmental guidelines on the use of PPE.

__________________________________________________________________

1. Are you satisfied with the quality of the PPE currently being used in your RT department?

i. For COVID 19 Positive/suspected patients

- 1. Yes
  2. No (why? Please Specify:_________________)

ii. For all other patients

1. Yes
2. No (why please specify)
3. Does your department have a ‘hot linac’ for confirmed COVID 19 POSITIVE patients?
   1. Yes
   2. No
   3. Not sure

Comments (Please specify)________________

1. Has your department implemented a ‘split teams’ procedure to limit the number of staff in the department at any one time?
   1. Yes
   2. No
   3. Not sure

Comments (please specify)___________________

1. Has your department implemented any of the following measures to minimise transmission? (tick all that apply)
   1. Perspex screens
   2. Separate entrance for confirmed COVID 19 patients
   3. Not sure/

Comments(please specify)_________________________

1. Has your department staggered appointment times to limit number of patients in waiting areas
   1. Yes
   2. No
   3. Not sure

Comments (please specify) ______________________

1. Is there an opportunity for treatment planning staff to work remotely?
   1. Yes
   2. No
   3. Not sure

Comments (please specify)

21. Is there an opportunity for treatment radiation oncologists to work remotely?

- 1. Yes
  2. No
  3. Not sure

Comments (please specify)

22. Is there an opportunity for medical physicists to work remotely?

1. Yes
2. No
3. Not sure

Comments (*please specify*)__________________________

**SECTION 3: Education and Training**

1. Did you receive specific education/training on correct PPE procedure during COVID-19 crisis?
2. Yes
3. No
4. I received general training prior to the COVID-19 crisis
5. Not sure

Other (please specify)_______________

1. There is evidence that COVID-19 may be detected on CBCTs in patients that were otherwise asymptomatic. Have you detected any suspicious lesions during routine clinical practice?
2. Yes
3. No
4. Not sure

Comments (please specify)_________________
